# Physicians and Specialties in the Veterans Health Administration’s Community Care Network

**DOI:** 10.1001/jamanetworkopen.2024.10841

**Published:** 2024-05-13

**Authors:** Yevgeniy Feyman, Kevin N. Griffith, Allison Dorneo, Sandra F. Simmons, Christianne L. Roumie, Kristin M. Mattocks

**Affiliations:** 1 US Department of Health & Human Services, Washington, DC; 2 VA Boston Healthcare System, Jamaica Plain, Massachusetts; 3 Department of Health Policy, Vanderbilt University Medical Center, Nashville, Tennessee; 4 Department of Health, Law, Policy and Management, Boston University School of Public Health, Boston, Massachusetts; 5 VA Tennessee Valley Health Care System, GRECC, South Nashville, Tennessee; 6 Department of Medicine, Vanderbilt University Medical Center, Nashville, Tennessee; 7 UMass Chan Medical School, Worcester, Massachusetts; 8 VA Central Western Massachusetts Healthcare System, Leeds

## Abstract

This cross-sectional study of data from the US Veterans Health Administration examines the availability of services provided through community care networks by specialty and clinical characteristics.

## Introduction

Increasing veterans’ access to care is a key policy priority for both the US Congress and Veterans Health Administration (VHA).^1^ Under the Maintaining Internal Systems and Strengthening Integrated Outside Networks Act of 2018 (MISSION Act), veterans may access care in the community, at VHA expense, provided they meet certain requirements such as an extended appointment wait or drive time for VHA care.^2,3^ To operationalize the MISSION Act, VHA contracted with Optum and TriWest, allowing veterans access to their health care networks and reimbursing their clinicians at Medicare rates.^4^ Millions of veterans have accessed community care at an estimated cost of 25% of the VHA’s medical care budget in 2024.^1^ Our aim was to describe the specialty coverage and representativeness of the VHA community care network.

## Methods

In this cross-sectional study, we used VHA administrative data to identify physicians who participated in the VHA community care network in calendar year 2019. We identified physicians who submitted Medicare claims from the Centers for Medicare & Medicaid Services’ Provider Utilization and Payment Data and Quality Payment Program, and practice information from the Provider Enrollment, Chain, and Ownership System.

We calculated descriptive statistics for physicians who did and did not participate in the VHA community care network. Differences were assessed using standardized mean differences (SMDs). SMDs greater than 0.1 were considered statistically different. We then calculated overall and specialty-level network participation, defined as the number of physicians who participated in VHA community care divided by the total number of Medicare participating physicians.

This study was considered exempt by the VA Boston Healthcare System institutional review board, with a waiver of informed consent because research could not be practicably carried out without exemption. We followed the Strengthening the Reporting of Observational Studies in Epidemiology (STROBE) reporting guidelines. More details are available in the eMethods in Supplement 1.

## Results

Of the 768 254 physicians who submitted a Medicare claim in calendar year 2019, 442 508 (57.6%) participated in the VHA’s community care network (Table). Compared with Medicare-only physicians, VHA community care network physicians had similar years since medical school graduation (mean [SD], 22.4 [12.0] vs 22.6 [12.9] years; SMD, 0.02). Their Medicare patient panels were similar in terms of proportion of male beneficiaries (mean male beneficiaries, 139.2 [42.2%] vs 203.6 [41.3%]; SMD, 0.07), mean (SD) age (71.3 [5.2] vs 71.3 [6.3] years; SMD, 0.01), mean (SD) hierarchical condition category risk adjustment score (1.7 [0.8] vs 1.7 [0.8]; SMD, 0.01), and mean (SD) Medicare payments per beneficiary ($343.6 [558.3] vs $359.6 [695.4]; SMD, 0.03). However, community care physician participants were more often male (67.2% vs 60.5%; SMD, 0.14), practiced in nonmetropolitan areas (13.7% vs 9.3%; SMD, 0.14) or health care professional shortage areas (27.8% vs 20.6%; SMD, 0.17), had larger Medicare patient panels (mean [SD] beneficiaries, 436.5 [690.3] vs 285.3 [471.8]; SMD, 0.26), and higher Merit-Based Incentive Payment System (MIPS) scores (84.7 [17.0] vs 82.3 [20.1]; SMD, 0.13).

**Table.** Characteristics of Physicians by Community Care Network Participation, 2019

Characteristics	Participants, No. (%) (n = 442 508)	Nonparticipants, No. (%) (n = 325 746)	SMD
Physician			
Male sex	297 523 (67.2)	197 155 (60.5)	0.14
Time since medical school, mean (SD), y	22.4 (12.0)	22.6 (12.9)	0.02
Practicing in nonmetropolitan areas^a^	60 479 (13.7)	30 358 (9.3)	0.14
Practicing in health care professional shortage areas^a^^,^^b^	76 691 (27.8)	34 441 (20.6)	0.17
MIPS final score, mean (SD)^b^	84.7 (17.0)	82.3 (20.1)	0.13
MIPS score >75^b^	228 247 (82.8)	129 042 (77.3)	0.14
Beneficiary^c^			
No. of total beneficiaries, mean (SD)	436.5 (690.3)	285.3 (471.8)	0.26
Age, mean (SD), y	71.3 (5.2)	71.3 (6.3)	0.01
Annual payments per beneficiary, mean (SD), $	343.6 (558.3)	359.6 (695.4)	0.03
HCC risk score, mean (SD)	1.7 (0.8)	1.7 (0.8)	0.01
Male sex, mean, No. (%)^d^	139.2 (42.2)	203.6 (41.3)	0.07

Abbreviations: HCC, Hierarchical Condition Category; MIPS, Merit-Based Incentive Payment; SMD, standardized mean difference.

^a^ Based on physicians’ primary practice locations.

^b^ This information is only available for MIPS participants (325 517 [42.3%]).

^c^ Characteristics are based on each physician’s panel of Medicare beneficiaries.

^d^ Beneficiary gender breakdowns were not reported for 10.7% of physicians (82 465).

Network participation was highest among oncologists (77.6%), urologists (77.3%), and cardiologists (76.3%) (Figure). Participation was lowest for nutrition (31.0%), speech pathology (31.3%), and emergency medicine (34.1%). Additionally, 41.2% of specialists in mental health and 51.9% of primary care physicians participated in community care.

**Figure.**
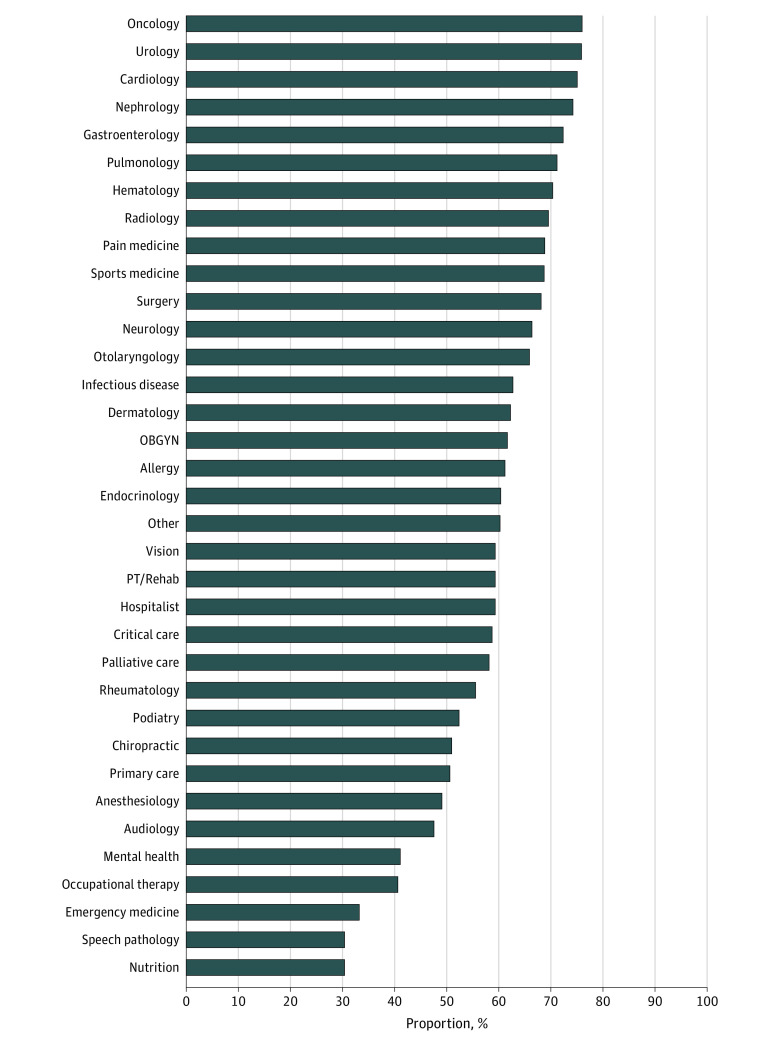
Specialty-Level Variation in the Proportion of Physicians Participating in the Veterans Health Administration’s Community Care Network, 2019 Specialty categorizations are further described in the eTable in Supplement 1. Percentages indicate the share of Medicare-participating physicians that also participated in the Veterans Health Administration community care network during calendar year 2019. OBGYN indicates obstetrics/gynecology; PT, physical therapy.

## Discussion

In this cross-sectional national analysis, we found that just over half of Medicare-participating physicians opt into the VHA community care network, albeit with specialty-level variation. Physicians participating in VHA community care were more likely to practice in rural and health care professional shortage areas where patients more often experience access barriers.^5^ Notably, participants had higher MIPS scores than nonparticipants, ameliorating concerns that the community care network would not attract high-quality physicians.^6^

Our results suggest that combined with the VHA’s existing workforce, veterans have broad access to high-quality community clinicians. However, our analysis has several limitations; our data do not indicate whether certain physicians nominally participate in the community care network but do not actively see veterans as patients, we cannot observe beneficiary characteristics for Medicare Advantage enrollees, and we cannot assess network adequacy. Future research should examine whether the VHA’s community care network is adequate to meet the care needs of US military veterans.

## Notes

Supplement 1.
eMethods.
**eTable.** Physician Specialty Categorizations **eFigure.** Sample Construction Flowchart

Supplement 2.
Data Sharing Statement
