# Supplementary material for: Physicians and Specialties in the Veterans Health Administration’s Community Care Network
Source: JAMA Netw Open. 2024 May 13;7(5):e2410841. doi: 10.1001/jamanetworkopen.2024.10841 (PMC11091756; doi:10.1001/jamanetworkopen.2024.10841)
Supplement: Supplement 1. — eMethods. eTable. Physician Specialty Categorizations eFigure. Sample Construction Flowchart [file jamanetwopen-e2410841-s001.pdf]

Supplemental Online Content

Feyman Y, Griffith KN, Dorneo A, Simmons SF, Roumie CL, Mattocks KM. Physicians and specialties in the Veterans Health Administration's community care network. *JAMA Netw Open.* 2024;7(5):e2410841. doi:10.1001/jamanetworkopen.2024.10841

eMethods.

eTable. Physician Specialty Categorizations

eFigure. Sample Construction Flowchart

This supplemental material has been provided by the authors to give readers additional information about their work.

© 2024 Feyman Y et al. *JAMA Network Open.*

eMethods.

Additional Notes on Data Sources
We used the VHA's Corporate Data Warehouse (CDW) and Provider Profile Management System (PPMS) to identify physicians who participated in the VHA community care network as of December 31st, 2019. Participation was defined as having an active contract with the VHA and did not depend on having VHA enrollees as patients. The PPMS data also indicated whether the physician was accepting new patients. We limited our sample to physicians with a valid national provider identifier (NPI) listed in the National Plan and Provider Enumeration System (NPPES) Registry. We identified providers who submitted at least one Medicare claim in 2019 from the Centers for Medicare & Medicaid Services' (CMS) Provider Utilization and Payment Data and Quality Payment Program. This data source also included data on characteristics of both physicians and their panels of Medicare beneficiaries. CMS assigns patients to physician panels based on Medicare claims received during that year. Additionally, we obtained Quality ratings and practice size data for clinicians participating in CMS' Quality Payment Program (QPP). Provider practice location was obtained from the Provider Enrollment, Chain, and Ownership System (PECOS) which identified the states in which each provider is licensed to practice medicine.

Physicians were the primary population and were identified by NPI. We limited our sample to only physicians within the 50 states and District of Columbia, excluding U.S. territories. Advanced practice providers were excluded because specialty is not identified for many of these clinicians. We also excluded specialties where expertise was provided without a patient encounter or the specialty was not for adult medicine: pathology, radiology, pediatrics, pediatric specialty care. We categorized physicians into 34 distinct specialty categories; specialties with fewer than 1,000 providers nationwide were grouped as "Other" (eMethods Table). A sample selection flow chart is provided in the eMethods Figure.

Detailed Description of Study Variables
Our primary outcome was network breadth, defined as the number of physicians who participated in VHA community care divided by the total number of Medicare participating physicians. Physician characteristics included specialty, gender, state(s) of licensure, year of medical school graduation, which is a correlate of number of years in clinical practice, practice size (number of clinicians), whether the physician is located in a rural area or healthcare professional shortage area, and final score under the Merit-based Incentive Payment System (MIPS). Physicians' MIPS scores range from 0-100, with higher scores denoting better overall performance in each of four categories (quality, cost, improvement activities, and promoting interoperability). We also identified physicians who received positive payment adjustments under MIPS, defined as a final score greater than 75.

Medicare Patient characteristics included mean age, percent male, average payments per beneficiary-year, mean Hierarchical Condition Category (HCC) risk score, and the total number of Medicare beneficiaries seen by the physician. HCC risk scores are a measure of projected future health spending based on patient demographics, prescription drug categories, and if comorbidities are present. The mean Medicare patient has a risk score of 1.0; patients that are healthier than average will have an HCC score below one and those that are less healthy than average would have a score above one.

Analytic Approach
Our unit of analysis was the individual physician. We linked Medicare and VHA data by physician NPI. We described the characteristics of physicians and patients who did and did not participate in the VHA community care network. Differences between the two groups were assessed using standardized mean differences (SMDs). A SMD value of less than or equal to 0.1 was considered an indication of similarity

© 2024 Feyman Y et al. *JAMA Network Open.*

while a SMD of greater than 0.1 indicated that differences between the two groups were unlikely to have occurred by chance.

© 2024 Feyman Y et al. *JAMA Network Open.*

eTable. Physician specialty categorizations

Specialty	Category
Allergy/Immunology	Allergy
Anesthesiology	Anesthesiology
Qualified Audiologist	Audiology
Adult Congenital Heart Disease	Cardiology
Advanced Heart Failure and Transplant Cardiology	Cardiology
Cardiac Electrophysiology	Cardiology
Cardiovascular Disease	Cardiology
Interventional Cardiology	Cardiology
Peripheral Vascular Disease	Cardiology
Chiropractic	Chiropractic
Critical Care (Intensivists)	Critical Care
Dermatology	Dermatology
Emergency Medicine	Emergency Medicine
Endocrinology	Endocrinology
Gastroenterology	Gastroenterology
Hematology	Hematology
Hematopoietic Cell Transplantation and Cellular Therapy	Hematology
Hospitalist	Hospitalist
Infectious Disease	Infectious Disease
Clinical Psychologist	Mental health
Clinical Social Worker	Mental health
Geriatric Psychiatry	Mental health
Psychiatry	Mental health
Psychologist Billing Independently	Mental health
Nephrology	Nephrology
Neurology	Neurology
Registered Dietitian or Nutrition Professional	Nutrition
Obstetrics/Gynecology	Obstetrics/Gynecology
Occupational Therapist in Private Practice	Occupational Therapy
Gynecological Oncology	Oncology
Hematology/Oncology	Oncology
Medical Oncology	Oncology
Radiation Oncology	Oncology
Addiction Medicine	Other
Dentist	Other
Medical Toxicology	Other
Nuclear Medicine	Other
Osteopathic Manipulative Medicine	Other
Other (Physician)	Other
Sleep Medicine	Other
Undersea And Hyperbaric Medicine	Other
Otolaryngology	Otolaryngology

© 2024 Feyman Y et al. *JAMA Network Open.*

Family Practice	Primary care
General Practice	Primary care
Geriatric Medicine	Primary care
Internal Medicine	Primary care
Preventive Medicine	Primary care
Physical Medicine and Rehabilitation	PT/Rehab
Physical Therapist in Private Practice	PT/Rehab
Interventional Pain Management	Pain Medicine
Pain Management	Pain Medicine
Hospice/Palliative Care	Palliative Care
Podiatry	Podiatry
Neuropsychiatry	Psychiatry
Pulmonary Disease	Pulmonology
Diagnostic Radiology	Radiology
Interventional Radiology	Radiology
Rheumatology	Rheumatology
Qualified Speech Language Pathologist	Speech Pathology
Sports Medicine	Sports Medicine
Cardiac Surgery	Surgery
Colorectal Surgery (Proctology)	Surgery
General Surgery	Surgery
Hand Surgery	Surgery
Maxillofacial Surgery	Surgery
Micrographic Dermatologic Surgery	Surgery
Neurosurgery	Surgery
Oral Surgery	Surgery
Orthopedic Surgery	Surgery
Plastic And Reconstructive Surgery	Surgery
Surgical Oncology	Surgery
Thoracic Surgery	Surgery
Vascular Surgery	Surgery
Urology	Urology
Ophthalmology	Vision
Optometry	Vision

© 2024 Feyman Y et al. *JAMA Network Open.*

eFigure. Sample Construction Flowchart

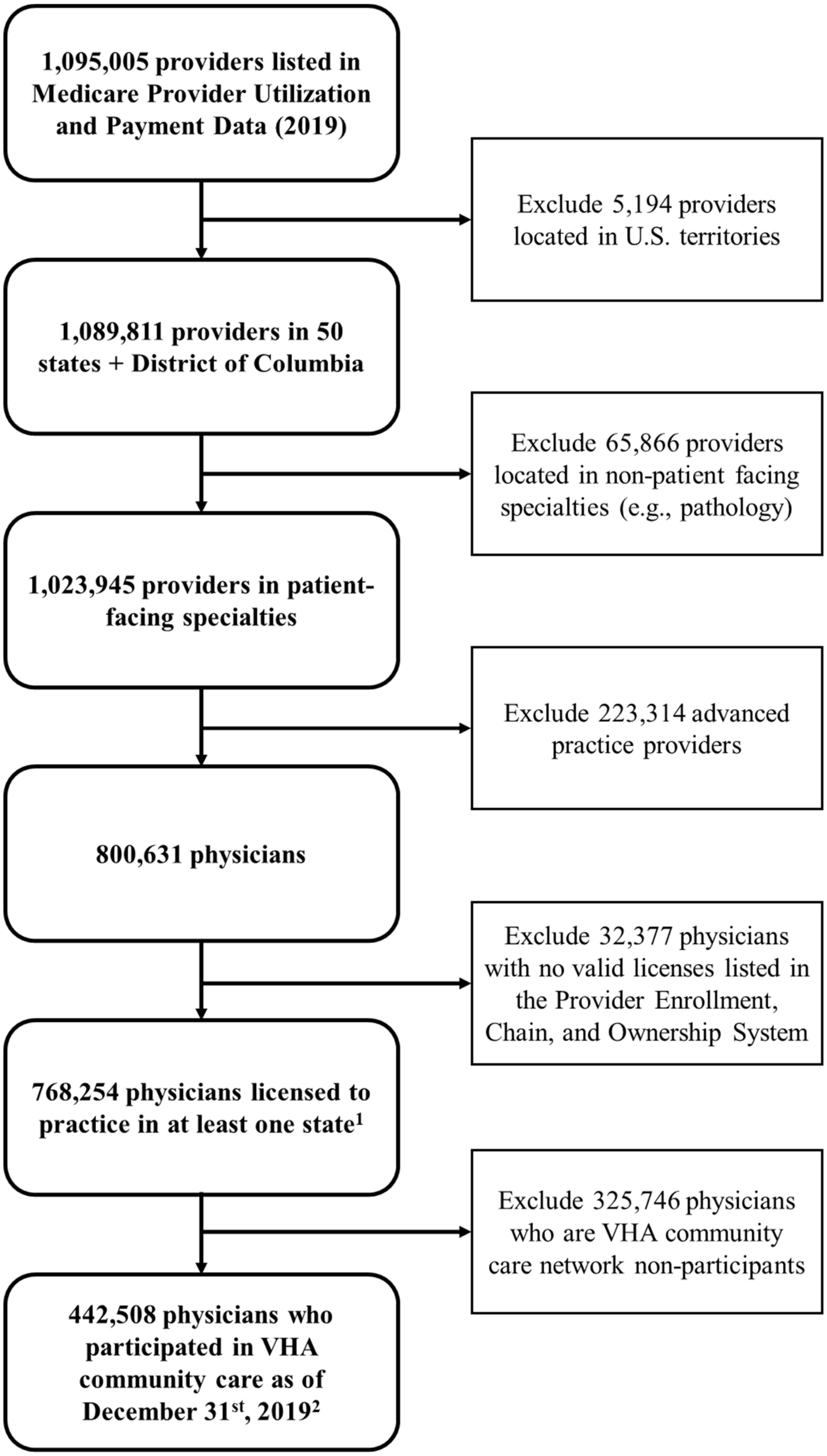

Notes: [1]This comprises our sample of Medicare-participating physicians. [2]This comprises our sample of participating physicians in the VHA community care network.

© 2024 Feyman Y et al. *JAMA Network Open.*
