# Supplementary material for: Physicians and Specialties in the Veterans Health Administration’s Community Care Network
Source: JAMA Netw Open. 2024 May 13;7(5):e2410841. doi: 10.1001/jamanetworkopen.2024.10841 (PMC11091756; doi:10.1001/jamanetworkopen.2024.10841)
Supplement: Supplement 2. — Data Sharing Statement [file jamanetwopen-e2410841-s002.pdf]

Data Sharing Statement

Feyman. Physicians and Specialties in the Veterans Health Administration's Community Care Network. *JAMA Netw Open*. Published May 13, 2024.
doi:10.1001/jamanetworkopen.2024.10841

Data
Data available: No

Additional Information
Explanation for why data not available: Aggregate data will be made available, but the VA does not allow posting of administrative data.
